# Measure to manage – an integrated pest management metric for horticulture

**DOI:** 10.1002/ps.70761

**Published:** 2026-03-26

**Authors:** Jennifer Byrne, Robert Lillywhite, Fiona Thorne, Lael Walsh, Henry Creissen

**Affiliations:** ^1^ Horticulture Development Department Teagasc Ashtown Research Centre Dublin 15 Ireland; ^2^ School of Life Sciences University of Warwick Coventry UK; ^3^ Rural Economy & Development Centre Dublin 15 Ireland; ^4^ Agronomy and Agriculture Institute University of the Highlands and Islands Kirkwall UK

**Keywords:** crop protection, IPM landscape, IPM systems, quantifying IPM, UK horticulture, vegetable and fruit production

## Abstract

**BACKGROUND:**

Integrated pest management (IPM) is the primary global crop protection paradigm. Based around a broad range of techniques, it is a framework for the management of pests (animal pests, weeds, diseases) with the reduction of pesticides as a core function. For policy, research and technological stakeholders to effectively assess and support IPM programmes, it is necessary to quantify adoption at the farm level. This study quantifies adoption in four temperate countries (Ireland, Northern Ireland, Scotland and England) and explores potential influence from the wider systemic landscape. Quantification is through the application of a peer‐reviewed horticultural IPM metric.

**RESULTS:**

The average IPM score for the four countries analysed was 58 of 100 (range 5–100). Growers from England averaged highest (64 of 100) and displayed strong levels of adoption of the core IPM practices, suggesting that there are elements within the IPM landscape in England contributing to the systemic supports required for higher adoption at the farm level.

**CONCLUSION:**

This exploratory study is the first to apply a universal metric for horticulture to a multinational dataset. The assessment of results on a regional basis allows for the comparison of IPM adoption from national to farm level, establishing a transferable framework for longitudinal benchmarking. We conclude that the overarching operational realm in which growers function has the potential to positively influence how IPM is adopted in the field and that the context most favourable to success is informed and cognizant about IPM motivations and limitations at the grower level. © 2026 The Author(s). *Pest Management Science* published by John Wiley & Sons Ltd on behalf of Society of Chemical Industry.

## Graphical abstract

IPM metric for food horticulture applied to growers in **four countries**.

Overall average score = 58/100.

**Top fruit** growers averaged highest (*M* = 61).

Growers from **England** scored highest overall & indicated strong adoption of core components

- Policy is an influencing factor, evidenced by adoption rates in England
- Policy must function at farm level to succeed at national level
- Market assurance schemes can support adoption

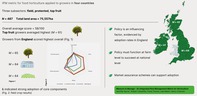

## 1 INTRODUCTION

Integrated pest management (IPM) refers to a method for managing harmful organisms, emphasizing a range of nonchemical approaches to minimize the use of, and risks associated with, pesticides.^1^ IPM as a construct is complex, multilayered and iterative,^2^ placed within a context of wide variance which includes crop type and production system.^3^ Over the decades of its existence, IPM has extended its defining boundaries. From an exclusively tactical strategy for the management of invertebrate pests, weeds and diseases^4^, ^5^ through various updated versions of the same, to being inclusive of modern technologies,^6^, ^7^ it has been envisioned as an entirely more expansive construct, incorporating broader agroecosystem considerations and cognizance of the principal pillars of sustainability.^8^ Although IPM is mainly a food production paradigm focused on the optimization of crop health, it also is an ecological paradigm.^9^, ^10^, ^11^, ^12^ IPM in commercial food production should, ideally, combine the benefits of chemical control with the minimization of risk^13^ through optimal synergy of its various components.^14^, ^15^ The literature is abundant with evidence for the ecological externalities attributable to unnecessary or excessive chemical application. These include negative impacts on air and water quality,^16^, ^17^, ^18^, ^19^, ^20^ destabilization of soil health,^21^, ^22^ toxicity to vertebrates and invertebrates including key crop pollinators,^23^, ^24^, ^25^, ^26^ and potential impacts on the health of the pesticide user and the end‐consumer.^27^

IPM is susceptible to farm‐level interpretation, as dictated by a complex multiplicity of spatial, temporal and grower‐specific factors.^28^, ^29^, ^30^, ^31^, ^32^, ^33^, ^34^, ^35^, ^36^, ^37^ Crop and production systems dictate the range and suitability of tactics.^38^, ^39^, ^40^, ^41^ Adoption is therefore not a binary practice/not practice concept as is sometimes depicted.^42^, ^43^ Rather, it is fluid and dynamic,^44^, ^45^ representing a continuum on which growers advance as their levels of integration^46^, ^47^ and competence develop. As such, the notion of quantifying IPM adoption is challenging.^48^, ^49^, ^50^ There is an abundance of literature on the adoption of horticultural IPM^51^, ^52^, ^53^, ^54^ and specific horticultural IPM practices,^55^, ^56^, ^57^ with the evidence providing insights into the motivations, limitations and perceptions of growers towards IPM.^45^, ^58^, ^59^, ^60^, ^61^ However, methodologies for assessing IPM uptake are often inadequate owing to arbitrariness as to which practices constitute IPM and inconsistencies in applied weightings, both of which can create misrepresentation of adoption levels.^62^ In reality, IPM is implemented in accordance with localized management concerns^63^ including pest pressure, ecological factors and landscape considerations,^64^ which draws attention to its nuanced and context‐specific nature and the risk of misrepresentation implicit in generic measurement approaches.

There are multiple benefits to measuring IPM practice including the ability to benchmark placement on a contextualized continuum, and to compare performance with that of preceding years and peers to establish future IPM goals. The literature shows that growers often look to their peers for information or reassurance before implementing IPM‐related changes in their practice,^65^ making benchmarking a source of motivation and an agent for change.^66^ Quantifying adoption provides insight into the effectiveness of systemic mandates which is essential owing to the level of investment and resources placed on IPM.^67^, ^68^ For many regions, including the entirety of the European Union (EU) and the UK, IPM is not merely one pest management strategy–it is the predominant pest management strategy.^69^ In measuring IPM, therefore, we can use grower adoption scores as a proxy indicator of the success or otherwise of elements within the wider IPM landscape, eliciting feedback on a complex concept within which multiple factors may be at play. Identifying the ways in which adoption is successful in one country is beneficial to other countries too, through the provision of reference points around best practice and routes towards uptake.^70^ In this way, robust metrics can serve as a device towards transnational collaboration.^71^

This study looks at the vegetable and fruit sectors of four European countries: Ireland, Northern Ireland, Scotland and England. In the context of this research, we include vegetable and fruit crops grown for human consumption. Mushrooms were excluded as a consequence of their distinctive production method. The four countries share broadly similar temperate climates and are prone to weather variability, although eastern and southern areas tend to enjoy drier and warmer conditions.^72^ The western and northern areas are characterized by a damper, moister climate even during the peak production months of July and August.^73^ Market contexts are comparable in that they follow the major retailer model and share a number of the main retail chains; however, Ireland is distinct in the extent of its market consolidation, having five main retail operators in comparison to approximately 10 in the UK. Membership of a quality assurance scheme is a prerequisite for access to the major retailer sales route in each of the four countries. In Ireland, the primary option is the Bord Bia Sustainable Horticulture Assurance Scheme (SHAS). In the UK, there are multiple schemes including those operated directly by the retailers, independent schemes and international schemes. Some of the UK retailers request membership of more than one. Horticultural food production is of high value across the entire geographical region of study (Table 1).

**Table 1:** Synopsis of the horticultural sectors for the four countries studied, based on 2023 and 2024 national censuses

	Total area (ha)	Value of sector	Regulatory instrument
Ireland	12 666^136^	€429 million^137^	National Action Plan^101^/Directive 2009/128/EC^1^
Northern Ireland	5177^138^	£156 million^139^	No formal instrument in place
Scotland	40 205^140^	£410 million^141^	Whole Farm Plan^84^
England	101 032^142^	£2.9 billion^142^	Sustainable Farming Incentive scheme^78^

In contrast to the similarities in climatic, agronomic and market factors, horticultural producers in the four countries operate under a range of IPM policies and frameworks, with varying degrees of statutory support and prescription. IPM in Ireland falls under the auspices of the EU as implemented at a Member State level. An updated and extended application of IPM for the EU was proposed through the formalization of the 2009 Sustainable Use Directive^1^ in the form of the Sustainable Use Regulation^74^ and in line with the initiatives incorporated into the Farm to Fork strategy.^75^ The European Parliament formally rejected this proposal in 2024 and the Sustainable Use Regulation was dismissed and withdrawn.^76^ Its current status remains unclear and at a Member State level, nations, including Ireland, continue to abide by the 2009 Directive. The devolved administrations in the UK take individual responsibility for their sustainable use of agricultural pesticides. England has progressed its agenda through the Voluntary Initiative (an industry‐led programme with many members including the Chemical Regulation Division and UK Governments^77^) and the Sustainable Farming Incentive (SFI)^1^ scheme in which growers are financially rewarded for a number of IPM‐based actions, one of which is ‘Assess integrated pest management and produce a plan’.^78^ Under this agreement‐level action, growers must assess and annually reassess their crop pest management strategies, with a view to planning how to most effectively incorporate IPM.^79^ Online assessment plans and planning tools are available for growers to utilize.^80^, ^81^, ^82^ One is the Voluntary Initiative IPM Plan, which guides growers through a range of questions on their current IPM tactics before providing detailed feedback and recommendations.^82^ Another is the IPM Planning Tool, which provides crop‐specific guidance for farm‐level pest management issues, enhanced by links to robust and independent research.^83^ The latter was built to complement the former, both attempting to bridge the knowledge gap cited by farmers as an impediment to increased adoption.^81^ As part of its 2025 Whole Farm Plan, the Scottish administration requires growers (or any business utilizing plant protection products) seeking to access Basic Payment (BPS) to complete several actions, one of which is an IPM plan.^84^ The BPS is a financial entitlement scheme based on the amount of land farmed and activities undertaken. For growers not currently in possession of an IPM plan both the English and Scottish administrations recommend a template^82^, ^85^ based on a validated and peer‐reviewed model for measuring IPM in temperate arable crops.^50^ There is no legislative framework for IPM in Northern Ireland although growers are strongly encouraged to complete one of the IPM plans referenced above. Overall, the UK Government recognizes the need for conscious IPM planning in order to improve and measure adoption. The process of planning, assessing and reassessing provides an opportunity for growers to gauge their longitudinal IPM progress, thereby restricting any ambiguity around IPM accomplishments,^86^ at farm and stakeholder levels.^87^

This study measures IPM adoption through the attribution of a unique score to a respondent body based in four countries. While there are divergences in the policy landscapes between the countries, there is sufficient similarity in an agronomic and market sense for insight to be gained from comparing the data. In other words, the relatively level playing field in terms of horticultural and sales factors allows for the exploration of other potentially influencing factors. The purpose of this study, therefore, is three‐fold: (i) to adapt and apply a peer‐reviewed IPM metric for horticulture^88^ to a multinational dataset. The metric will be adapted through a straightforward reapportioning process to accommodate available common data; (ii) establish the extent and type of IPM adoption across the four countries; and (iii) reflect on the macro IPM landscape in which growers (as the end users of IPM) operate.

## 2 METHODS

### 2.1 The survey data

Two distinct surveys were used to collect data from four countries. One was designed by the National Farmers' Union (NFU) and Voluntary Initiative (VI), based on a template originally created for the measurement of IPM in arable crops^50^ and intended for UK (Northern Ireland, Scotland and England in this context) growers.^82^ A version of this survey was tailored specifically for Scottish growers and had the involvement of the Scottish Plant Health Centre (formally known as Scotland's Centre of Expertise for Plant Health).^85^ A second survey, also based on the Creissen *et al*.^50^ model, was designed by Teagasc and the University of Warwick, and intended for circulation amongst Irish growers.^88^

The surveys were designed and used independently, yet they shared an identical motive and a number of common questions–six in total. This commonality allowed for the results of both data collection processes to be combined into one substantial dataset. The six questions common to all data are listed in Table 2 and are the core components on which the IPM scoring process was conducted–in effect, the performance indicators embedded within the metric. In the case of both surveys, the participants were targeted through a probability approach, wherein horticultural food producers were informed of and invited to respond via social media, pest bulletins, mailing lists, and at in‐person fora such as grower events and sector meetings. The UK survey was open for completion over a 2‐year period from 2022 to 2024 and the Irish survey over 5 months in 2023. Respondents to both survey instruments were self‐selecting in the sense that there was no financial or other incentive to participate during the time of data collection. This suggests that there may be an inherently positive bias towards IPM within the sample. Although we believe there is no clear sample selection bias attributable to the agencies responsible for data collection, each engaging with growers in a similar capacity, the authors recognize that growers who are more aware of, or more directly engaged with, the advisory institutions and their networks involved in collecting the data may be more likely to participate in the research, making this sample more representative of a particular sample of growers instead of the entire grower population.

**Table 2:** Core metric questions common to the two survey instruments used to collect data on IPM adoption from Ireland, Northern Ireland, Scotland and England

Metric Question 1:	What factors do you consider when choosing crop variety (and rootstock where relevant)?
Metric Questions 2–4:	For this cropping year, which management measures will you employ to prevent/control pests on the land that you farm or manage: To prevent/control invertebrate pests? To prevent/control disease? To prevent/control weeds?
Metric Question 5:	What factors will you consider when developing and evaluating your IPM plan?
Metric Question 6:	Are you a member of crop association or crop discussion group?

### 2.2 The IPM metric for horticulture

The horticulture metric was developed from an arable metric,^50^ which employed a Delphi^89^, ^90^ group consensus approach to create a weighting structure for a series of key IPM components. The arable metric was novel for two main reasons; first, it was based on a robust calculation of ratings and weightings of IPM components relative to their importance in an IPM programme which was, second, founded entirely on opinion of IPM practitioners (advisors and growers). The metric was constructed around a series of core IPM practices, each assigned a weighting out of 100 based on the relative value of the core practice to an overall IPM programme or plan. Within each of the questions were a range of subcomponents pertaining to the specific practice. These were rated on a scale of 1 to 5, with 1 being the lowest and 5 being the highest score achievable. The arable metric has become the instrument of choice for quantifying IPM within the UK, routinely used by farming organizations (e.g. NFU), quality assurance boards (e.g. Red Tractor) and government departments (e.g. DEFRA). It also has been widely endorsed by the farming community, with almost 17 000 farmers utilizing the metric through a range of IPM planning tools since its launch in 2020.^81^ The horticulture metric extended the arable model by adapting the core IPM components to food horticulture and establishing a weighting structure utilizing an expanded Delphi‐style approach.^88^ The weighting structure was separated according to the horticultural subsectors of field, protected and top fruit crops to cater for and incorporate a sensitivity to the divergence within crop production systems, crop‐specific agronomic considerations and pest‐specific management choices. A compound score incorporating the three subsectors was then calculated. Two steps were involved in calculating the score, as follows: (1) a total score was calculated for each of the core metric questions, including all subcomponents; and (2) the question‐level scores were combined into an overall IPM score for the subsector. The methodology is described in detail in Byrne *et al*.^88^ The maximum score achievable was 100.

The original horticulture metric contained eight core IPM components for field crops, seven for protected crops and six for top fruit crops, as relevant to the unique systems of production. Of the core IPM components^2^ in the original metric, six were common to the full dataset for the four countries. In practice therefore, the top fruit weightings remained unchanged, the field and protected weightings required adaptation. A simple reapportioning technique ensured that the integrity of the original Delphi‐defined weightings was unaltered. For example, in the original context, metric question 1 for field crop growers weighted 12.2; when reapportioned out of 100 to reflect the reduced number of common core questions for the four‐country dataset, this adjusted to 15.7. The weighting structure herein therefore deviates from that reported in Byrne *et al*.^88^ Table 3 provides the final reapportioned weightings applied to the core metric questions.

**Table 3:** Weightings applied to the core metric questions, reapportioned out of 100 to reflect data common to the full dataset

Metric questions	Field	Protected	Top fruit
MQ1. Factors considered when choosing crop variety	15.7	22.1	22.5
MQ2. Preventative measures to control the introduction & spread of invertebrate pests	21.4	24.6	17.5
MQ3. Preventative measures to control the spread of disease	19.1	20.4	26.0
MQ4. Preventative measures to control weeds	16.0	5.7	6.8
MQ5. Factors influencing decisions when developing pest management plan for the year	14.5	20.2	16.3
MQ6. Membership of grower/ crop discussion group	13.3	7.0	11.0
**Total**	**100**	**100**	**100**

### 2.3 Data analysis

Application of the IPM metric to the dataset was conducted in IBM spss statistics v29 data analytics software (IBM Corp., Armonk, NY, USA). A normality test was conducted to assess the distribution of the data; outliers were assessed using the IQR 1.5 rule which states that any score 1.5 times above the upper quartile bound or below the lower quartile bound is an outlier. Survey responses were categorized under one of three subsectors (field, protected, top fruit) and further categorized in accordance with the predominant crop type. This approach enabled the examination of correlations between categorical variables such as crop and IPM component or subcomponent, comparison between the three subsectors to the subcomponent level and, comparison across the four countries. Cross‐tabulations were conducted to assess the impact of a range of variables on IPM score, for instance membership of an assurance scheme. One‐ and two‐way ANOVAs were performed throughout the analysis to compare the effect of subsector and nationality on overall IPM score. *Post hoc* Tukey's honestly significant difference (HSD) tests were used to establish the degree of significance where subsector and nationality differences were identified.

## 3 RESULTS

### 3.1 Sample and sociodemographic results

Table 4 provides a summary of respondent data (*n* = 487) by subsector and by country, as well as the land areas covered by the respondent farms and their estimated representative percentage of total land areas (TLA). Not all respondents provided their land areas and therefore the figures provided below should be interpreted as minimum. UK crops grown outdoors in containers were included under ‘field’ for the purposes of calculating land areas: Ireland has no equivalent. Ireland and England provided data on the three subsectors, and Northern Ireland and Scotland on two with the exclusion of protected and top fruit, respectively.

**Table 4:** Summary of the number of respondents by subsector and country, plus the minimum land areas (ha) covered by the respondents with the estimated representative percentages of TLA

	Field	Protected	Top fruit	Totals
Ireland	65	23	12	100
	4542 (38%)	142 (57%)	348 (61%)	5032
Northern Ireland	15	0	7	22
	1621 (41%)	0	240 (19%)	1861
Scotland	32	22	0	54
	10 616 (28%)	914 (47%)	0	11 530
England	155	72	84	311
	50 899 (50%)	657 (33%)	5578 (26%)	57 134
Totals	267	117	103	**487 respondents**
	67 678	1713	6166	**75 557 ha**

### 3.2 Results of the application of the IPM metric

**Figure 1:**
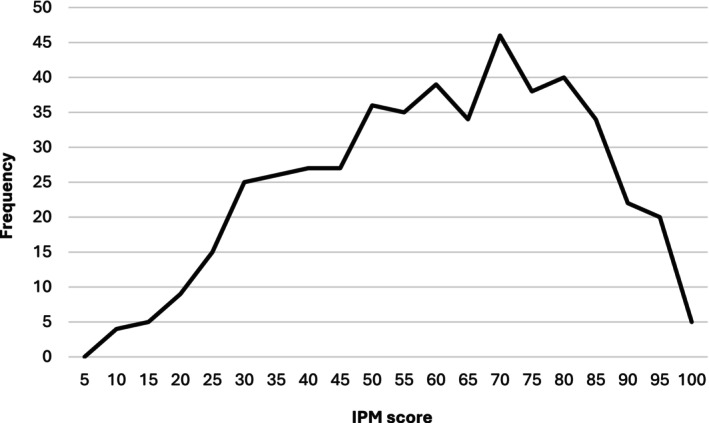
Distribution of scores – the IPM continuum – for the full dataset.

The data were normally distributed, and no outliers were identified (Supporting Information, Fig. S1). Across the dataset, the average IPM score was 58, with a minimum score of 5 and a maximum of 100. As the three subsectoral weighting systems shared a common maximum of 100 IPM points, it was possible to generate a single IPM continuum for the entire respondent body across the four countries (Fig. 1). Growers from England achieved an overall *mean IPM score* of 64, followed by Northern Ireland (mean = 55), Ireland (mean = 47) and Scotland (mean = 42).

**Figure 2:**
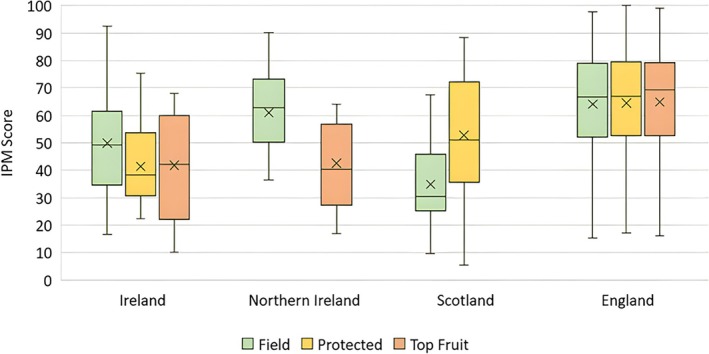
Box‐and‐whisker plot illustrating the IPM score for each subsector, by country. The median (‘___’) and mean (‘X') are highlighted to illustrate data distribution.

At a subsector level, the top fruit cohort scored higher in their mean value (mean = 61) than the protected (mean = 58) and field crop growers (mean = 57) respectively, although a one‐way ANOVA showed there was no significant difference between the IPM means for the three subsectors. The data were subsequently examined for difference between subsectoral means by country. Figure 2 includes the subsectoral medians and means to illustrate data distribution, which showed that field crop growers from Scotland were lowest scorers overall (mean = 35), followed by protected growers from Ireland (mean = 41) and top fruit growers from Ireland and Northern Ireland (mean = 42 and 43, respectively). The sample sizes by country (Table 4), being varied, may have had some impact on these scores. Internally, the greatest similarity in subsectoral means was displayed by growers from England and, to a lesser extent, Ireland.

**Figure 3:**
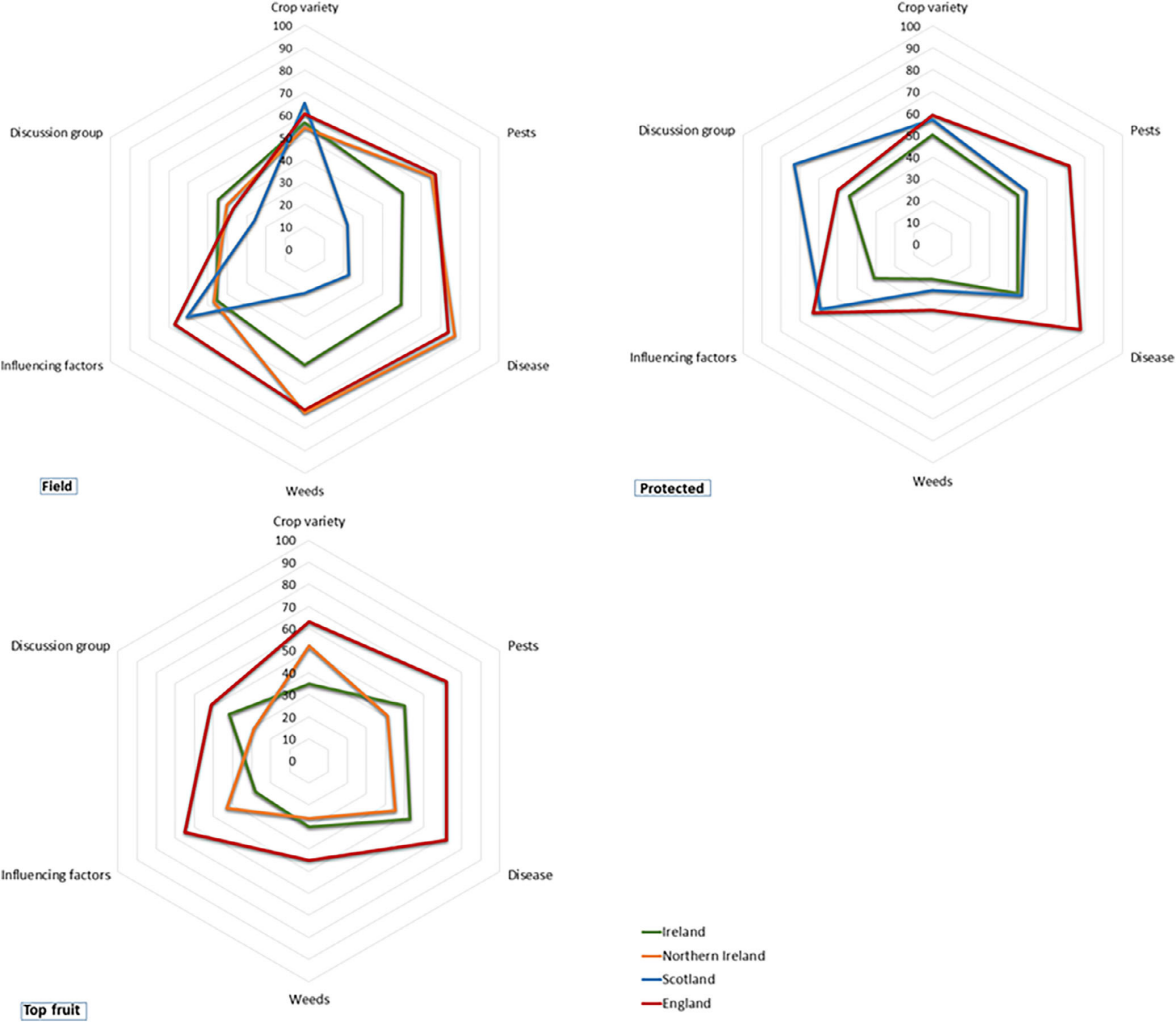
Mean IPM scores for each of the core metric questions, by subsector and country. The mean scores are presented as a percentage of the total score achievable for each metric question and normalized from 0 to 100 for comparative purposes.

The six core metric questions were analyzed for levels of adoption and compared by subsector and country. Figure 3 presents the mean IPM score for each metric question by subsector and country as a percentage of the total value attributed to the specific metric question. The mean scores have been normalized from 0 to 100 in order to capture their relative rate of adoption.

Across the six, core field metric components (Table 5), field growers from England achieved the highest mean IPM score (mean = 64), followed by Northern Ireland (mean = 61), Ireland (mean = 50) and Scotland (mean = 35). The mean score for Scotland was significantly lower than all other national mean scores (*P* < 0.001 for England and Northern Ireland, and *P* = 0.001 for Ireland) and the mean score for Ireland was significantly lower than that for England (*P* < 0.001) but not for Northern Ireland (*P* = 0.163). Figure 3 shows that field crop growers from Northern Ireland and England were similar in mean IPM scores for crop variety, invertebrate pest, weed and disease management, and membership of a discussion group. They displayed divergence in relation to influencing factors (that is, the factors influencing pest management plan decisions), growers from England achieving higher mean rates of adoption. The field growers from Scotland showed highest mean adoption of crop variety, also scoring relatively highly on influencing factors and low on the remaining metric‐based practices. Field growers from Ireland showed greatest consistency in their mean component scores. They displayed lower rates of adoption on the invertebrate pest, weed and disease components and influencing factors than their peers from Northern Ireland and England, relatively close rates of adoption of crop variety practices and highest adoption of discussion group membership.

**Table 5:** Overall mean IPM scores for each country, by subsector, with SD and SEM

	Field			Protected			Top Fruit		
	Mean	SD	SEM	Mean	SD	SEM	Mean	SD	SEM
Ireland	49.9	19.1	2.4	41.4	14.5	3.0	41.8	20.7	6.0
Northern Ireland	61.0	16.6	4.3				42.6	17.1	6.4
Scotland	34.6	16.8	3.0	52.7	21.2	4.5			
England	64.0	19.0	1.5	64.4	19.6	2.3	64.9	19.5	2.1

Overall, the protected crop respondents from England (Table 5) achieved the highest mean IPM score (mean = 64), followed by Scotland (mean = 53, *P* = 0.033) and Ireland (mean = 41, *P* < 0.001). The protected crop growers from England (Fig. 3) surpassed their peers from Scotland and Ireland by a wide margin in their uptake of IPM techniques relating to invertebrate pest, weed and disease and by a narrow margin for influencing factors and crop variety. The protected crop growers from Scotland displayed the highest mean score for membership of a discussion group. The cohort from Ireland scored the lowest means across the core metric components particularly in relation to influencing factors and membership of a discussion group.

The highest mean IPM score (Table 5) across the core component practices for top fruit was achieved by England (mean = 65). Ireland and Northern Ireland were closely aligned with mean IPM scores of 43 and 42, respectively. England showed significant difference to both Ireland (*P* < 0.001) and Northern Ireland (*P* = 0.012). The top fruit growers from England (Fig. 3) achieved the highest mean IPM score across all core components, their highest mean scores related to invertebrate pest and disease management practices. The top fruit growers from Ireland followed with regards invertebrate pest and disease management scores, and also in relation to weed management and membership of a discussion group. Those from Northern Ireland displayed the weakest adoption levels in terms of invertebrate pest, disease and weed management, and membership of a discussion group, but surpassed Ireland in adoption of crop variety and influencing factor practices.

A number of additional variables were cross‐tabulated with IPM score by country for assessment of impact. Membership of an assurance scheme was cited by 83% (*n* = 405) of all respondents, which was not unexpected given that assurance scheme affiliation is an entry‐level requirement for access to the major retailer sector. Of those not affiliated with an assurance scheme, 43% (*n* = 35) grow on ≤1 haand 44% (*n* = 36) are organically certified; we surmise that these respondents utilize alternative routes to market such as farmers' markets, box schemes or community‐supported agriculture. For the UK respondents, Red Tractor was overwhelmingly the most commonly quoted assurance scheme although membership of several schemes was cited, including GLOBALG.A.P., LEAF Marque, Tesco Nurture and M&S Select Growers. In Ireland, growers were asked about their membership of SHAS. By country, Northern Ireland displayed the highest level of membership at 95% (*n* = 21), followed by England (90%, *n* = 281), Scotland (84%, *n* = 46) and Ireland (57%, *n* = 57). Within the upper quartile of scorers from the four countries, 94% (*n* = 115) have assurance scheme membership contrasting with 67% (*n* = 82) of the lower quartile.

## 4 DISCUSSION

This exploratory study reports on the application of an IPM metric to a combined dataset from the food horticulture sectors of Ireland, Northern Ireland, Scotland and England, providing an opportunity to adapt and extend a novel metric^88^ for horticulture. The key innovation within the methodology is its ability to measure IPM performance at the subsectoral level (field crop, protected crop, top fruit) using a single composite metric. This facilitates the assessment of IPM adoption levels in a nuanced manner, sensitive to the differences in agronomic practices and pest threats. The process of adapting the existing weightings allowed for the confirmation of the metric's applicability to alternative data, and the approach to adaptation proved uncomplicated. One of the strengths of the original metric was the substantial involvement of growers (the expert IPM practitioners) in its development, both in terms of guidance around the activities to be measured and the weight of measurement to be applied to those activities. Not all survey instruments will be identical, therefore the benefit of having a universal metric^87^ is substantiated in this context in the sense that four national datasets mirrored each other through a multilateral reflection of what constitutes IPM.

The mean IPM scores indicate that the respondents from England are the highest IPM adopters from the four countries, followed by Northern Ireland, Ireland and Scotland. Earlier research on arable IPM reported similar findings, although in that study England was followed by Scotland, Ireland and Northern Ireland.^50^, ^91^ The IPM continuum created by the results shows that adoption is widespread, based on the premise that even a low IPM score demonstrates some level of adoption.^49^ The results also show that it is indeed possible for growers to achieve the maximum score of 100. However, the mean scores imply that adoption levels can be improved across the nations and subsectors. This suggests that the ‘average’ grower represented in the dataset is, potentially, forfeiting the opportunity to take full advantage of the IPM options applicable to them. It also suggests that while IPM is being implemented across the four countries, the degree to which it is being implemented is inconsistent; future research is necessary to further quantify this inconsistency. There are wide differences, for example, in subsectoral means for Northern Ireland and Scotland. We surmise a combination of crop pathogen considerations and limitations in corresponding IPM options in the case of top fruit growers in the former and field crop growers in the latter. The unique and valuable Northern Ireland Bramley apple industry is vulnerable to apple scab (*Venturia inaequalis*),^92^ which appears to be under‐resourced in terms of decision support^93^ and therefore demands the largest share of annual fungicide applications^94^ for the subsector. The narrow margin for error in managing apple scab is evidenced in this study by the very low adoption score for disease by the cohort. The majority of the field growers from Scotland cited potato as their predominant crop. Late blight (*Phytophtora infestans*) is an issue in the Scottish climate and a robust fungicide programme is recommended by extension services as the primary defence protocol.^95^ Again, very low adoption scores for disease are displayed by this cohort. However, these instances represent just two particular pockets of practice with niche crop issues which do not entirely account for the broader inconsistency. Only the respondent body from England display subsectoral similarity in uptake (Ireland does to a lesser extent) and a mean adoption score of >60. This suggests that there are broader provisioning factors supporting IPM adoption in England. It is therefore meaningful to consider the wider IPM landscapes in which the growers within the four countries operate.

Lefebvre *et al*.^96^ subdivide European public policies towards IPM into three types, namely regulatory instruments, measures to disseminate information, and incentive instruments such as subsidies or taxes. The SFI scheme for England is an amalgam of these categories. As it pertains to IPM, the SFI has been in place since 2023.^3^ Within the scheme, there are four funded IPM‐based actions, one of which is the completion of an IPM assessment and plan (option CIPM1),^79^ to be reassessed on an annual basis. This action is supported by a number of planning templates.^82^, ^83^ Current evaluation figures state that 15 800 farmers and growers have committed to an agreement under this option, as well as 1700 agreements under CIPM2 for the development of flower‐rich areas, 2100 agreements to companion crop on arable and horticultural land (CIPM3) and 8000 agreements to not use insecticide on arable or permanent crops (CIPM4).^97^ Completion of one of the recommended IPM plans provides immediate and evolving access to current, field‐tested research^80^ for managing specific invertebrate pests, diseases and weeds. Respondents also are granted a real‐time assessment of their IPM performance with the potential to stimulate adoption through the provision of a personalized benchmark of progression; this is in line with psychological theories of social comparison.^98^, ^99^ According to stakeholder feedback from early pilot studies on the SFI, the scheme has the potential to raise grower consciousness around IPM, while completion of one of the IPM assessment plans has the capacity to increase commitment to IPM compared with existing practice levels.^100^ However, growers in Ireland are currently functioning in an IPM vacuum. The withdrawal of the Sustainable Use Regulation^74^, ^76^ means that Irish IPM remains operational under the conditions of the 2019 National Action Plan (NAP)^101^ which holds little or no legal weight or incentivization. With no incentive‐based measures in place to encourage adoption or incremental improvement, horticultural IPM in Ireland is at a clear disadvantage compared to the UK. The European Court of Auditors, in their report on the progress of implementation of the 2009 Sustainable Use Directive^102^ identified assessment of IPM adoption as an area requiring improvement. They concluded that Member States, including Ireland, needed to establish performance indicators for IPM uptake to allow for the monitoring of implementation of the SUD and associated adjustment of strategy. The stagnation and ensuing uncertainty connected with the lack of a strong policy driver is likely to hinder growers in their development and progression along the IPM continuum; Lane makes a similar conclusion in a comparison of EU and US IPM approaches.^103^ Although Northern Ireland's growers have been operating without a legal IPM framework, they have been exposed to the IPM assessment plans for some time, implying that they may have benefitted from similar influence as their colleagues in England and Scotland. Future investigation is required to establish the causation for the low performance of the field growers from Scotland; we have alluded to the pathogen/action limitation in relation to late blight as an example, which raises the question as to whether research, extension and policy are sufficiently provisioning growers in their IPM progression. This cohort is currently in preparation for the Whole Farm Plan,^84^ through which BPS is contingent on the completion of a range of audits and plans. For horticultural producers in particular, this includes a biodiversity audit, carbon audit, soil analysis and an annual IPM plan. It will be interesting to reassess their IPM performance in future years to establish effectiveness of this combined suite of elements.

Continuing their promotion of IPM, the English government has recently introduced a much anticipated Pesticides National Action Plan (NAP).^104^ This plan incorporates a Pesticide Load Indicator (PLI)^105^ based on 20 metrics, four relating to environmental fate and 16 to ecotoxicity. The development of the UK PLI provides a potentially historic shift towards the integrated analysis of pesticidal impact on a range of key environmental and ecological indicators but it is, in its nascent form, an instrument to document pesticide usage trends. The UK PLI is based on the Danish PLI^106^ and has some commonality with the German version of the Total Applied Toxicity (TAT) indicator.^107^, ^108^ Denmark introduced ambitious pesticide actions in the 1980s with the development of the Treatment Frequency Index (TFI)^109^ coinciding with support and incentivization for a shift towards organic (pesticide‐free) food production through the Organic Farming Act in 1987. The TFI was based on the number of doses of pesticide applied, with no differentiation made for high or low toxicity load.^110^ It did not have the desired long‐term effect on pesticide reduction and was replaced by the PLI taxation system in 2013, although it continues to be the primary pesticide use indicator in France.^111^ The Danish model is a case study in pesticide reduction success^110^ which has been reinforced by a strong emphasis on the provision of research, advice, demonstration and decision support services. In providing annual pesticide usage data to the administration, Danish growers contribute to a complex model capable of identifying areas of ecological vulnerability owing to pesticide load. This, in turn, provides growers with invaluable agronomic information.^112^ As such, the Danish system facilitates substantiated agronomic decision‐making which has proven to be effective in reducing pesticide load overall, despite some reported crop‐specific limitations.^110^ Norway also has a differentiated pesticide taxation system^113^ which is enhanced by extensive knowledge support efforts.^114^, ^115^ Under this framework, cereal growers were found to be generally high achievers in IPM adoption.^49^ A secondary benefit is the motivation of pesticide manufacturers and distributors to provide alternative products of lower toxicity.^113^

It is unclear as to the future intentions for the UK PLI and the extent to which it will unfold. Caution must be exercised to avoid any reversal in the IPM adoption gains made to date in England. The recent pause on new members to the SFI, subject to budgetary reassessment,^116^ has caused disquiet in the farming media around the disruption to agri‐environmental progress^117^ and farm planning.^118^ Growers function in the complex space between agroecosystem and food system, with farm system economic viability a central concern, particularly when there is uncertainty around subsidization. It is incumbent upon them to fulfil multiple commitments to a range of stakeholders, often in the face of conflicting interests.^40^ Within our study, there is a substantial cohort of growers meeting their food provisioning commitments under low IPM adoption levels, suggesting that there is little immediate impetus for them to progress their IPM development. To capture and drive the interest in IPM progression of these individuals, therefore, account must be taken of the personal and situational heterogeneity of farm‐level IPM by the wider IPM systemic landscape. Without the type of supporting instruments noted by Lefebvre *et al*. and provisioned in the English context, this cohort of growers can continue to operate in an IPM *status quo*, which presents a loss to the potential to elevate IPM beyond the farm level. In addressing farm‐level IPM, elements within the macro landscape raise the opportunity for far‐reaching impact on area‐wide ecological infrastructures beneficial to IPM;^119^ for instance, shelter habitats as a tool towards enhanced conservation biological control.^120^ In this sense, farm‐level niches of practice can contribute to scaled IPM which has the potential to become a delimited, self‐reinforcing system.^121^

The market environment in which growers operate also has a function as a driver of IPM adoption.^122^, ^123^, ^124^ For example, a UK‐wide review of the farm assurance system^125^ asked if assurance schemes are an essential requirement; compared to other agricultural sectors, horticultural producers responded the most positively. This outcome implies that horticultural producers see a valuable role for assurance schemes beyond access to the major retailer supplier route. At a minimum, Irish growers subscribe to the assurance scheme Bord Bia SHAS, within which there is a requirement to maintain a statutory IPM record^126^; in a sense, SHAS reinforces the existing NAP.^101^ In the UK, conversely, there are multiple assurance schemes, some of which demand a high‐level adoption of IPM by growers. The Red Tractor Fresh Produce Standards^127^ was the most commonly cited scheme in this research by a wide margin. Following strong criticism of its role in and oversight of IPM implementation,^128^ Red Tractor now requires growers to complete an IPM plan such as the VI IPM Assessment Plan. The distinction between countries is that Irish growers continue to abide by a dated set of IPM standards, lacking in reflection of current techniques, research and development. On the contrary, the UK‐based IPM assessment plans and Planning Tool are integrated with regulation in a live and evolving manner, driven by the desire of growers to develop their IPM skills.^100^ Growers have expressly stated the benefit of integrating statutory compliance and assurance scheme mandates; indeed the latter is seen as a driver of the former.^129^ Future research is required to explore drivers and barriers to assurance scheme membership beyond route to market and whether factors such as farm size or production system are influential.

The extension of the model in this study to a cross‐national context allows for investigation and comparison of what is possible in countries with similar IPM structures, potentially improving adoption rates by example. Networking IPM on a multilateral basis has been identified as an efficient use of resources, pooling personnel and data towards a common goal.^130^ It is known that the larger commercial growers involved in this study engage in cross‐border communication with their peers working under analogous systems. This takes the form of informal networks and attendance at conferences and industry events. It is also known that the larger Irish growers of high‐value specialist crops rely on the expertise of overseas agronomists and biological control consultants.^88^ Two points emerge: first, policy‐ and extension‐level resources might be shrewdly targeted in enabling direct engagement and knowledge exchange as a means to enhance the general IPM learning environment.^130^ This has potential benefit not only to the large commercial enterprises, but also to those growers for whom accessibility may be limited owing to scale.^34^ Projects such as IPMWorks,^131^ which provide Europe‐wide farmer networking and demonstration opportunities, have initiated progress in this direction. Second, this study highlighted a notable weakness around membership of grower/crop discussion groups, with the exception of protected crop growers in Scotland who operate within a long‐standing area of soft fruit production. Further research will be required to establish the causation for the low membership of grower/crop discussion groups; for the Ireland and Northern Ireland situations, it may, in part, be attributable to the small population sizes and concerns around competitiveness and trust.^132^ Given the benefits associated with these types of groups^71^, ^124^, ^133^, ^134^ policy should encourage their formation, the case of Scotland's protected fruit subsector potentially providing useful insight; Denmark provides another template through their system of farmer experience groups,^134^ and the US promotes communication and collaboration through Regional IPM Centers.^124^ Additionally, Scottish–indeed all UK–growers of protected crops have access to Producer Organizations (POs), of which there are approximately 32 in the UK overall. By contrast, horticultural POs in Ireland are an underdeveloped source of opportunity, with only a handful of organizations representing the larger scale growers, mushroom production being the most significantly represented crop. This denies small‐ or medium‐scale Irish growers the prospect to partake of the benefits of PO membership, one of which is the facilitation of knowledge transfer and innovation.^135^

## 5 CONCLUSION

The original IPM metric for horticulture^88^ proved adaptable across a four‐country dataset, suggesting its applicability to a wider range of horticultural settings. The metric has the potential, through its ability to capture crop‐specific nuance, to serve as an evolving tool in the longitudinal assessment of IPM and effectiveness of external, systemic‐level drivers into which much state and research resources are directed. Its capacity to operate across different countries provides the opportunity for international benchmarking and dialogue; at the farm level, it can serve as a self‐reflection tool in the development of the IPM practitioner (the grower) and localized indicator of the effectiveness of policy, research and technological elements. In summary, our findings imply that the systemic IPM landscape in which growers operate has an effect at the farm level. The most successful systemic landscapes reinforce IPM adoption through a range of elements including grower‐supporting regulations, economic‐based incentives, and/or research and advice networks. There also is a case to be made for improved integration of market assurance schemes with policy initiatives. Of the four countries studied in this research, growers from England were the most motivated to optimize their IPM adoption through the strong incentivization provided by elements of the wider environment.

Further research is required to investigate the causes behind the subsector‐ and country‐specific differences in IPM score and adoption of the core IPM activities evidenced by the findings. This study has established an IPM baseline for the four countries represented, but to contribute to the reduction of pesticides and the economically sustainable and ecologically sound management of crop health, greater understanding of the motivating and limiting factors at farm level is imperative.

## FUNDING INFORMATION

This research was funded by the Teagasc Walsh Scholarship Programme (scholarship no. 2021047).

## CONFLICT OF INTEREST

The authors declare no conflict of interest.

## Supporting information


**Figure S1.** Normal probability–probability plot indicating that the distribution of scores over the IPM metric key variables was normal.

## Data Availability

The Irish survey is reported in Byrne *et al*., 2025.^88^ The UK survey is available to access through https://www.nfuonline.com/updates-and-information/fill-in-your-ipm-plan/#menu. Survey data for England, Scotland and Northern Ireland was obtained under a data sharing agreement with the National Farmers' Union, the James Hutton Institute and the University of Warwick. The Irish survey data is available under reasonable request.

## Footnotes

^1^
The SFI ceased to accept new applications as of March 2025. All existing or in‐process agreements are to be honoured. An ambitious and long‐delayed UK Pesticides National Action Plan was launched in March 2025, incorporating a Pesticide Load Indicator. A reformed SFI scheme will be launched mid‐2026.

^2^
The two other core questions included in the original IPM metric for horticulture but not common with the UK data and therefore excluded were: What proportion of your field outdoor cropping land is in continuous production for 3 or more consecutive years without growing a break crop? and What is the primary management reason for (this) rotation? The former was applicable to field crops only, the latter was applicable to field and protected crops.

^3^
The English growers in this dataset operate under the existing SFI agreements. Their agreements will not change with the implementation of a reformed scheme.
