# Supplementary figures and images for: Measure to manage – an integrated pest management metric for horticulture
Source: Pest Manag Sci. 2026 Mar 26;82(7):6817–28. doi: 10.1002/ps.70761 (PMC13240703; doi:10.1002/ps.70761)

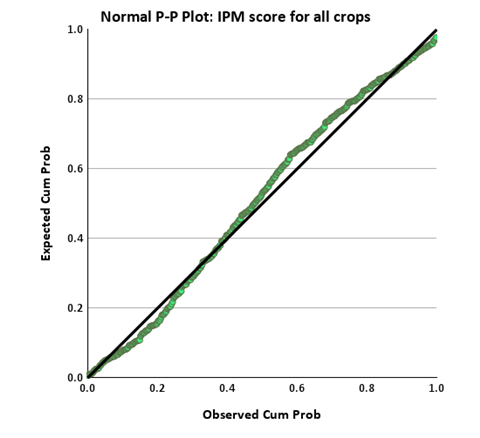

Supplement: Supplementary file 1 — Figure S1. Normal probability–probability plot indicating that the distribution of scores over the IPM metric key variables was normal. [file PS-82-6817-s001.png]
